# Polysaccharides and Structural Proteins as Components in Three-Dimensional Scaffolds for Breast Cancer Tissue Models: A Review

**DOI:** 10.3390/bioengineering10060682

**Published:** 2023-06-03

**Authors:** Eva Pasquier, Jennifer Rosendahl, Amalie Solberg, Anders Ståhlberg, Joakim Håkansson, Gary Chinga-Carrasco

**Affiliations:** 1 RISE PFI AS, Høgskoleringen 6b, NO-7491 Trondheim, Norway; eva.pasquier@rise-pfi.no (E.P.); amalie.solberg@rise-pfi.no (A.S.); 2 RISE Unit of Biological Function, Division Materials and Production, RISE Research Institutes of Sweden, Box 857, 50115 Borås, Sweden; jennifer.rosendahl@ri.se (J.R.); joakim.hakansson@ri.se (J.H.); 3 Sahlgrenska Center for Cancer Research, Department of Laboratory Medicine, Institute of Biomedicine, Sahlgrenska Academy, University of Gothenburg, 41390 Gothenburg, Sweden; anders.stahlberg@gu.se; 4 Wallenberg Centre for Molecular and Translational Medicine, University of Gothenburg, 41390 Gothenburg, Sweden; 5 Department of Clinical Genetics and Genomics, Sahlgrenska University Hospital, 41345 Gothenburg, Sweden; 6 Department of Laboratory Medicine, Institute of Biomedicine, University of Gothenburg, 40530 Gothenburg, Sweden; 7 Department of Chemistry and Molecular Biology, University of Gothenburg, 40530 Gothenburg, Sweden

**Keywords:** breast cancer models, biopolymers, 3D bioprinting, cells microenvironment

## Abstract

Breast cancer is the most common cancer among women, and even though treatments are available, efficiency varies with the patients. In vitro 2D models are commonly used to develop new treatments. However, 2D models overestimate drug efficiency, which increases the failure rate in later phase III clinical trials. New model systems that allow extensive and efficient drug screening are thus required. Three-dimensional printed hydrogels containing active components for cancer cell growth are interesting candidates for the preparation of next generation cancer cell models. Macromolecules, obtained from marine- and land-based resources, can form biopolymers (polysaccharides such as alginate, chitosan, hyaluronic acid, and cellulose) and bioactive components (structural proteins such as collagen, gelatin, and silk fibroin) in hydrogels with adequate physical properties in terms of porosity, rheology, and mechanical strength. Hence, in this study attention is given to biofabrication methods and to the modification with biological macromolecules to become bioactive and, thus, optimize 3D printed structures that better mimic the cancer cell microenvironment. Ink formulations combining polysaccharides for tuning the mechanical properties and bioactive polymers for controlling cell adhesion is key to optimizing the growth of the cancer cells.

## 1. 3D Cancer Tissue Models

Cancer is one of the most common diseases, with 19.3 million new cancer cases annually [1], with breast cancer as the most diagnosed and leading cause of cancer death among females [2]. There are several different treatment regimens for cancer available, including surgery, radiotherapy, and chemotherapy, but their efficacies differ between patients.

Cancer is the medical indication with the most new potential drug substances in the pipeline for clinical evaluation [3]. However, because of low conformity between the human cancer milieu and the in vitro systems used today for drug efficacy screening, the vast majority of all candidates fail in late clinical phases despite promising results in preclinical model systems [4,5]. For cancer drugs, only 5% of the preclinical candidates reach the market after demonstrating sufficient efficacy in phase III trials [5,6]. The U.S. Food and Drug Administration (FDA) and the European Federation of Pharmaceutical Industries and Associations (EFPIA) have independently examined the causes behind the decreasing productivity of successful drug candidates and identified that improvements in the predictivity of safety and efficacy will have the highest potential for reversing this negative trend [7].

Animal models are frequently used in cancer research. One common model is the xenotransplantation of human tumor cells into immunocompromised mice. Implanting primary tumor cells from patients with this technique is called patient-derived xenografts (PDXs) and has been reported from the 1970s for testing of new pharmaceutical agents [8,9,10]. PDX models of breast cancer tumors have, as an example, been used for the evaluation of the chemotherapy response [11,12]. However, the drawbacks with this model are that animal models are expensive, they are only possible to perform in small scale, and they suffer from low predictive value related to humans. This is due to microenvironments that consist of an animal, and not human, stroma, which is reflected in the drug effect on the cells. The animal stroma causes the cells to be more proliferative compared with a human stroma—resulting in a higher effect in response to antiproliferative agents. Another difference is that these animals are immune defect, which may affect the treatment response [5]. Importantly, legal requirements are increasing towards the use of alternative, non-animal models in the regulatory safety assessment of chemicals [13].

Tumor characteristics and development are still not perfectly understood, and there is a challenge in how to translate in vivo characteristics to in vitro models to help with understanding the tumors as well as developing new therapeutic agents [14]. The most commonly used in vitro models today are conventional two-dimensional (2D) cell cultures in plastic petri dishes. However, 2D plastic cultures do not resemble the human situation very well and influence a more differentiated and proliferative cell phenotype while the in vivo three-dimensional (3D) environment enriches it in terms of cancer stemness, cell-to-cell contact, migration, and a mixture of differentiation stages and cellular function [15,16,17]. Different 3D cell culture systems, including organoids [18], spheroids [19], and hanging drop arrays [20], have been developed to overcome the issue of limited cell-to-cell contact in a monolayer growth. In these models, cells can grow in multilayers, which facilitate cell-to-cell interactions in different directions. However, all current systems lack a microenvironment similar to the growth conditions in human tumors in vivo.

The aim with 3D cell culture systems is to mimic the microenvironment surrounding the cancer cells, also creating gradients of oxygen and nutrients. Every tumor is composed of cells that possess unique characteristics [21], the breast tissue is made up of different components, with mammary epithelial cells, stromal fibroblasts, and the extracellular matrix (ECM) being the most significant parts. The ECM predominantly contains collagens, heparin, laminins, glycosaminoglycans (GAGs), elastin, and fibronectin, creating the microenvironment [22]. To generate these 3D models, hydrogels are potential candidates as they have high water retention capacity and can generate soft and porous structures for the cells to grow in [23]. Three-dimensional printing and bioprinting are other strategies to produce 3D scaffolds as they have the advantage of tuning the shape and properties of the scaffold as well as incorporating cells within the structure when mixed with the ink before printing. Three-dimensional printed scaffolds can mimic human tumor tissue more closely compared with 2D cell cultures [24,25,26].

**Figure 1:**
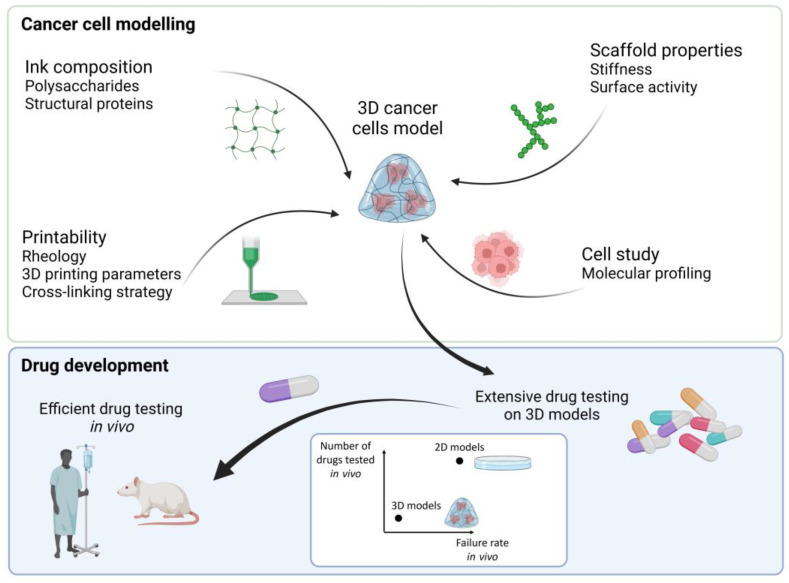
Steps to make a 3D cancer cell model by combining different biopolymers and cells to make a bioink for 3D printing. After optimization of the cancer cells microenvironment screening of high number of drugs is possible before selecting the most efficient drugs for in vivo testing, hence reducing the number and improving the success rate of drugs tested in vivo. Created with BioRender.com.

This review focuses on the preparation of 3D breast cancer tissue models made of natural polymers that can be 3D printed (Figure 1). For simplicity’s purposes, the natural polymers (made of biological macromolecules) are referred to as biopolymers in this review. We present properties related to 3D printing of polymers, and more specifically, hydrogels and the potential biopolymers used for the printing of 3D cancer tissue models. The biopolymers presented are divided into two groups: polysaccharides and bioactive polymers. The polysaccharides present high mechanical properties to support cell growth while bioactive polymers (structural proteins) have an adhesive surface that interacts with cells and facilitates cell attachment [27]. Importantly, we explore how relevant polysaccharides can be modified to become bioactive with improved properties that mimic the cancer cell microenvironment. Scaffold properties related to breast cancer cell growth are presented, and characterization with a gene expression assessment is highlighted.

## 2. Additive Manufacturing

Three-dimensional printing has become a powerful tool for constructing defined 3D architectures, including direct ink writing, melt extrusion, inkjet, selective laser sintering, stereolithography, digital light processing, and tomographic additive manufacturing. Three-dimensional printing provides reproducibility and control of the shape and pore size, although not all the technologies are adequate for bioprinting and tissue models. Interested readers should see the reviews on the topic [28,29,30].

Micro-extrusion (or also called direct ink writing) has been the one of the most applied 3D printing technologies. The technology is based on inks that are extrudable (i.e., required inks with adequate ink rheology/shear thinning). Hence, care has to be taken regarding the rheology of the applied ink as it may affect the pressure needed to extrude the bioink (cells can be added into the ink before printing), consequently affecting the forces applied on the cells and, thus, cell viability [31]. Since cells are sensitive to temperature and stresses, an adequate ink matrix must comply with some critical properties, including optimized rheology, gelability, and cross-linking ability.

**Figure 2:**
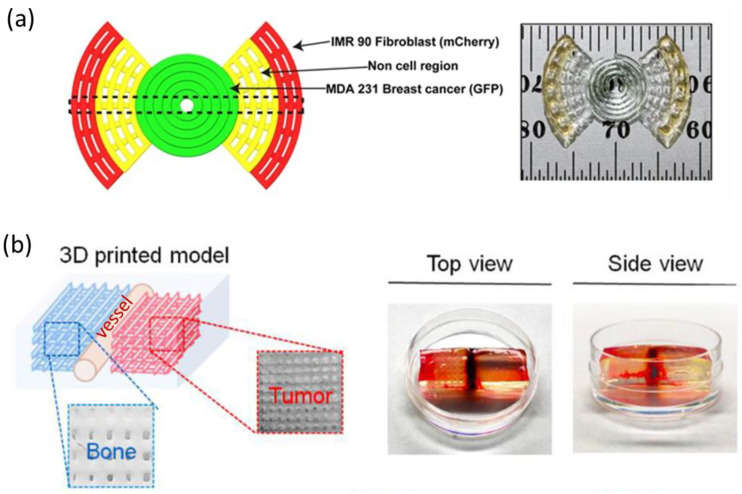
3D printing of complex structures for in vitro tumor modeling. (**a**) 3D bioprinting of a heterogeneous tumor model comprised of both MD Anderson-Metastatic Breast-231 Cells (MDA-MB-231) breast cancer cells and Institute for Medical Research-90 (IMR-90) fibroblasts to study cells migration and interactions. Model and photograph of the bioprinted sample. Reproduced with permission from [32] (**b**) 3D printing of a vascularized tissue model for studying breast cancer metastasis to the bone. Schematic of printed model and images of the bone and tumor regions. Images of 3D printed sample with top and side view. Reproduced with permission from [35].

Three-dimensional printing provides possibilities to make complex structures with different materials [32,33] and cell deposition in controlled locations [34] (core-shell structure) with good reproducibility (Figure 2). However, as it is based on hydrogels, micro-extrusion may yield poor shape fidelity of the 3D printed constructs. An alternative method was applied by Cui et al. (i.e., laser direct writing) where the authors reported the 3D printing of a model that represents cancer cells and bones separated by a vessel to observe the migration of the cancer cells to the bone cells. Nano-hydroxyapatite doped gelatin methacrylate/polyethylene glycol diacrylate (GelMA/PEGDA) and GelMA/PEGDA ink were used for the bone matrix and the cancer matrix, respectively, while the vessel part was printed with GelMA [35].

In addition to the previously mentioned 3D printing technologies, recent advances have demonstrated the potential of new additive manufacturing methods (i.e., tomographic volumetric additive manufacturing) [36]. This technology has been developed to overcome the geometric constraints and throughput limitations of layer-by-layer technologies such as micro-extrusion. Tomographic volumetric additive manufacturing is based on inks that in most cases are photopolymerizable by UV light. However, cell viability and proliferation can be affected by UV light intensity and the type and concentration of the photoinitiator [37].

To avoid the potential detrimental effects of UV light that are usually applied in photopolymerization, it could thus be most interesting to apply visible light and natural photo-initiators to polymerize 3D constructs [38]. Chiulan et al. [38] suggested Riboflavin (vitamin B2) as one of the natural photo-initiators that has been proven to be non-toxic, biodegradable, and biocompatible. Riboflavin has been used to cure furfuryl-alginate derivative [39] and collagenous biomaterials [40] with visible light.

### 2.1. Rheology of the Ink

Ink rheology is a key parameter for 3D printing by micro-extrusion as it determines the printability of the material. When the viscosity is too low, the yield stress might not be sufficient to support the weight of the material and the filaments fuse after printing, leading to poor shape fidelity [41]. In contrast, when the ink is too viscous, a clogging of the nozzle can happen, or the printing might not be continuous and may cause cell stress.

Depending on the type of material, the viscosity might be temperature or shear dependent. In any case, rheology needs to be assessed to determine the conditions of printing as well as the composition of the ink. Hydrogels with a shear thinning behavior are interesting, as they facilitate the 3D printing process. Viscosity of the ink under stress needs to be studied to determine suitable printing parameters, such as the ink concentration and printing speed. Fast elastic recovery and sufficient elastic modulus are also important to maintain the shape after printing. Cellulose nanofibers (CNF) present shear thinning behavior at low concentrations, at high shear stress disruption of the network and alignment of the nanofibers along the shear direction allow the gel to flow [42]. When the ink rheology is temperature-dependent, viscosity profile as function of temperature is crucial.

### 2.2. Cross-Linking Mechanisms

**Figure 3:**
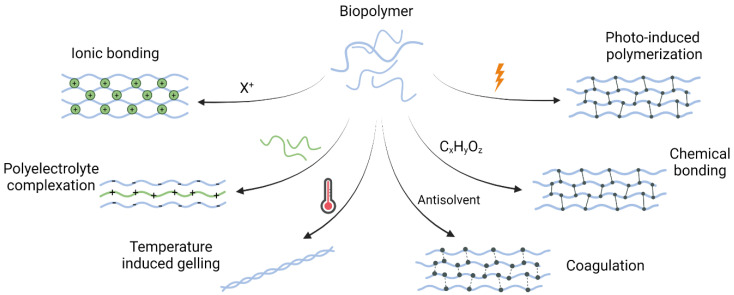
Different types of cross-linking commonly used to stabilize 3D printed biopolymer structures. Created with BioRender.com.

There are different ways to cross-link the structure of a 3D printed hydrogel scaffold, it can be performed during or after printing. The most common cross-linking methods are ionic cross-linking, chemical cross-linking, photo induced polymerization, temperature induced gelling, polyelectronic complexation, and coagulation in a non-solvent bath (Figure 3) [28]. The cross-linking method depends on the type of hydrogel and if the hydrogels are printed to form a scaffold before cell seeding or loaded with cells before printing (bioink) (Table 1). For example, alginate is commonly ionically cross-linked with a CaCl_2_ solution. Ca^2+^, which is a divalent cation, reacts with the carboxylic acid groups in alginates. Cross-linking is a crucial step, as it further influences the final properties of the scaffold. By tuning the CaCl_2_ concentration from 0.2 to 1 M, Cavo et al. obtained stiffnesses of alginate hydrogels varying from 150 to 4000 kPa [43]. If long time stability is needed, chemical cross-linking could be necessary, as biodegradation due to ion exchange could occur for ionically cross-linked polymers. For example, carboxylated CNF can be covalently cross-linked with amines due to the occurrence of aldehyde groups on the nanofibril surface [44]. Ajdary et al. combined polyelectrolyte complexation of carboxylated CNF and chitosan during 3D printing and chemical cross-linking with glutaraldehyde as a post-treatment to enhance the flexibility of the structure [45]. It is also possible to ionically cross-link negatively charged polymers with the growth media, as it contains several cations [46]. In that case, different mechanical properties are obtained depending on the growth media used [25].

### 2.3. Bioprinting

Three-dimensional bioprinting is a biofabrication process that uses bioinks (i.e., inks containing cells). It allows to have the cells directly distributed in the bulk of the hydrogel and not limited to the surface. With bioprinting, different types of cells can be printed in predetermined locations of the scaffold. Complex structures where different cells can migrate and interact with each other can be formed by this mean [32]. However, it also implies more constraints on the 3D printing parameters and cross-linking approach (Table 1), as cells are fragile and cannot tolerate high shears or temperatures. Temperatures from 4 °C to 40 °C have been reported; 40 °C seems to be the maximum temperature, as higher temperatures will denature proteins [47]. Another challenge is that the material can suffocate the cells if oxygen and nutrients cannot reach the cells (e.g., in human tissue the gas and nutrients diffusion capacity is limited to a depth of approximately 150 µm) [48,49,50].

The optimization of the viscosity of the ink for bioprinting is important as it needs to be viscous enough to maintain a good shape fidelity but liquid enough to be able to print at a reasonable pressure for the cells to survive (lower than 100 Pa) [28]. Ouyang et al. studied the viability of embryonic stem cells during the 3D printing of a gelatin/alginate hydrogel [51]. They found a correlation between the shear stress applied during 3D printing and cell viability. A cell viability greater than 90% was obtained when the shear stress was lower than 100 Pa. The range of viscosity associated with the pressure will depend on several parameters, such as the temperature, the type of material, and the shape of the nozzle.

Microbial contamination poses a significant challenge in the realm of a cell and tissue culture. Establishing and consistently maintaining aseptic practices holds the utmost importance, as a healthy cell culture is a prerequisite for obtaining accurate outcomes, particularly in applications like 3D bioprinting [52]. The material obtained from the manufacturer can be bought sterile, or the polymers can be sterilized after manufacturing by, for example, autoclaving, gamma-irradiation, or a chemical treatment. It is, however, important that the sterilization method does not affect the material characteristics, and careful consideration must be taken when choosing the sterilization method. For tissue-derived scaffolds, a chemical treatment with peracetic acid is one sterilization method that has been shown suitable for both in vitro [24,53,54] and in vivo [55,56] studies.

**Table 1:** Cross-linking strategies used for scaffold laden with cells after or during 3D printing.

	Ink Composition	Cross-Linking Method	Specific Type of Cross-Linker	Results	References
**3D printed scaffolds**	Ad-MeHA (adamantane-modified and methacrylated hyaluronic acid) and CD-MeHA (cyclodextrin-modified and methacrylated HA)	Guest-host cross-linking before printing (cyclodextrin-adamantane) and photo-induced cross-linking during printing (methacrylated HA)	Irgacure 2959 for photopolymerization of methacrylated HA, 5 min UV at 320−390 nm	Guest-host cross-linking was necessary for stable printing and covalent cross-linking was needed for long term stability. Structures were stable over one month. The methacrylate moieties allowed to chemically attach RGD motifs.	[57]
	CNF and gelatin	Temperature gelling (gelatin) during printing and chemical cross-linking (gelatin and genipin) post-printing	Genipin	Gelatin gel was mechanically reinforced with CNF; maximum strength was obtained with 10% of CNF. Cross-linking with genipin was completed within 24h and increases with genipin concentration.	[58]
	TEMPO CNF	Ionic cross-linking during printing and chemical cross-linking post printing	CaCl_2_ and, 1,4-butanediol diglycidyl ether (BDDE)	Compression modulus increased with the amount of cross-linker. The scaffold was stable for 3 months in PBS. Higher cross-linker amounts led to higher cell proliferation due to increasing stiffness of the scaffold.	[59]
	TEMPO CNF and alginate	Ionic cross-linking	CaCl_2_	Alginate reduced the print quality (form and shape factors are reduced). When cross-linked with CaCl_2_, alginate reinforced the CNF structure post-printing.	[60,61]
	Galactoglucomannan methacrylate (GGMMAs) and TEMPO CNF	Photo-induced cross-linking post printing	Irgacure 2959, 5 min UV at 320−390 nm	Compressive modulus was tuned depending on GGMMAs type and concentration. GGMMA was non cytotoxic and supported cell proliferation.	[62]
	Collagen and chitosan	Physical gelling (collagen) during printing, ionic and chemical cross-linking post-printing	NaOH and genipin	Degradation rate and mechanical properties were controlled by the chitosan concentration. Chitosan decreased the degradation rate of collagen and increased its mechanical properties.	[63]
	Chitosan and TEMPO CNF	Polyelectrolyte complexation during printing and chemical cross-linking post-printing	Glutaraldehyde	Mixture of TEMPO CNF and chitosan was not printable (not homogeneous ink). Multilayers of chitosan/TEMPO CNF and TEMPO CNF were printed in chitosan bath. A maximum of 10% weight loss was obtained after one month.	[45]
**Cell-laden scaffolds**	Pentenoate-functionalized hyaluronic acid (PHA), rBMSCs and rNSCs cells	Photo-induced and chemical cross-linking	Irgacure 2959 for UV cross-linking (312 nm for 2 min), dithiothreitol (DTT)	The cross-linking chemistry was fast with low amount of photo-initiator. rBMSCs had long term viability while rNSCs viability was affected by the bioprinting. High cell concentrations had minimal effect on the printed shape fidelity, yield stress, and viscosity.	[64]
	Gelatin, silk fibroin and hTMSCs cells	Enzymatic or physical (sonication) cross-linking	Mushroom tyrosinase or sonication	The swelling of enzymatically crosslinked structure was higher compared to the sonicated structure due to lower amount of β-sheets structure of silk fibroin. Enzymatically crosslinked structure was stable over one month while sonicated structure was stable for 7 days because of gelatin release.	[65]
	Gelatin/alginate/fibrinogen (G–A–F) or gelatin/alginate (G–A) and 293FT cells or Hela cells	Physical gelling (gelatin), ionic (alginate), and enzymatic cross-linking (fibrinogen)	CaCl_2_ and thrombin	Alginate brought time stability to gelatin structure. Ionic cross-link of alginate was more stable than the temperature crosslinking of gelatin. Fibrinogen was added to chemically stabilize the structure. The structure with fibrinogen was stable over 30 days of cell culture.	[31,51,66]
	Alginate and U87-MG cells	Ionic cross-linking	CaCl_2_ before and during printing and BaCl_2_ post-printing.	The stability of the structure was increased from 3 days to 11 days by adding a post-printing cross-linking with BaCl_2_. Cell viability was 93% after bioprinting and maintained over 88% after 11 days.	[67]

## 3. Biopolymers for Additive Manufacturing

**Figure 4:**
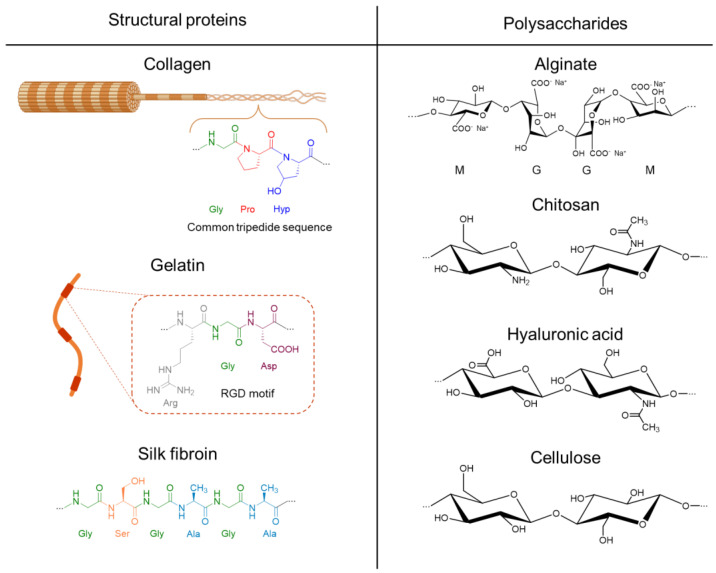
Structure of relevant proteins and polysaccharides that can form 3D scaffolds that support cancer cell growth: collagen, gelatin, silk fibroin, and alginate on the sodium form, chitosan, hyaluronic acid, and cellulose.

Biopolymers have been used to model cancer tissues and, more specifically, breast cancer tissues (Table 2) because of their high biocompatibility, bioactivity, and ability to form hydrogels. There are two major classes of biopolymers that can be used in cancer models: polysaccharides (alginate, chitosan, cellulose, hyaluronic acid) and proteins (gelatin, collagen, silk fibroin). Polysaccharides and proteins are considered structural and bioactive polymers, respectively (Figure 4). Each biopolymer has specific properties, and usually, a combination of different biopolymers is used to obtain a structurally stable hydrogel with a bioactive surface. Hence, it is common to find optimal combinations of, for example, alginate/gelatin [27], agarose/collagen [68], alginate/chitosan [69], alginate/hyaluronic acid [70], cellulose/gelatin [71], alginate/hyaluronic acid [72], and silk fibroin/chitosan [73].

Below, we provide an overview of the different biopolymers used in a breast cancer tissue model and their properties related to 3D printing and cancer cell growth. Other polymers, such as fibrinogen, agarose, and elastin, could be used as additives in 3D printing scaffolds, but they are not the main constituent of the structures [66,68,74,75].

**Table 2:** Examples of 3D printed scaffolds used for breast cancer tissue modeling. The following cell lines have been included: MD Anderson-Metastatic Breast-231 Cells (MDA-MB-231), Institute for Medical Research-90 (IMR-90) cells, Michigan Cancer Foundation (MCF) cells, epithelial cells (EpH4).

Hydrogel	Cell Line	3D Printing Technique	Results	Ref.
**Alginate/Gelatin**	MDA-MB-231 and IMR-90 (fibroblast cells)	Micro-extrusion	2 cell lines were printed at specific initial locations. Fibroblast migrated, infiltrated the MDA-MB-231 spheroids, and created mix MDA-MB-231/IMR-90 multicellular tumor spheroids.	[32]
**Alginate/Gelatin**	MDA-MB-231	Micro-extrusion	The hydrogels composition influenced bioprinting and cell adhesion. The rheology of different alginate/gelatin composition was studied. Increase in gelatin concentration led to higher cells proliferation and larger tumor spheroids.	[27]
**Collagen/Matrigel**	MDA-MB-231 and EpH4	Micro-extrusion	Collagen could not be 3D printed alone, Matrigel was added to improve its rheology. Collagen fiber alignment was controlled during 3D printing. Cancer cells oriented along the collagen fibers direction.	[76]
**Collagen**	MCF-12A, MCF-7 and MDA-MB-468	Micro-extrusion (injection of cells in collagen gel)	Chimeric (human mammary organoids with cancer cells) structures for cancer cells redirection by a normal microenvironment were formed. Efficiency of chimeric organoid formation was higher using bioprinting process (90% at 14 days) compared to manual matrix embedding procedures (<10%).	[77]
**Chitosan/gelatin**	MCF-7	Electrodeposition	Chitosan/gelatin 3D structures were coated with alginate to reach 7 days stability. Electrodeposited hydrogels were biocompatible, but cells did not spread.	[78]
**Alginate, hydroxyapatite and periostin**	MCF-7 and MDA-MB-231	Micro-extrusion	Mechanical properties of the scaffold were tuned depending on the alginate concentration. MCF-7 and MDA-MB-231 showed different cellular adhesion and proliferation behavior. Similar drug response was obtained between 3D printed alginate scaffold and patient derived scaffold.	[24]
**TEMPO CNF**	MCF7 and MDA-MB-231	Micro-extrusion	TEMPO CNF formed porous 3D structures suitable for cancer cells growth. The cell culture media influenced the scaffold mechanical properties. The expression of genes related to stemness, and migratory properties were increased compared with 2D cultures.	[25]

### 3.1. Collagen

Collagen is the most abundant component of the extracellular matrix (ECM) of many tissues, including skin, bone, tendons, and blood vessels [23]. There are several types of collagens; in humans, type I, III, and IV are present, among others. Collagen I is the most abundant in humans representing 90% of all collagen [79]. It has three polypeptide chains arranged in a triple helix structure, which can self-assemble into fibrils (see Figure 4). Moreover, the polypeptide chains contain the RGD (Arg-Gly-Asp) motif that promotes cell adhesion. Collagen gelifies at 37 °C, but the gelation is slow, and the mechanical properties and time stability of the gel are poor, which is not suitable for 3D printing [68]. Moreover, due to a low shape retention, pure collagen may be cross-linked [80] or combined with polysaccharides, such as alginate, agarose, chitosan, or CNF, to improve its stability and printability [63,68,74,81].

Chen et al. reported that the growth of MCF-7 cells in a 3D cross-linked collagen structure resulted in longer cell proliferation time and the overexpression of pro-angiogenic growth factors compared with 2D cell cultures [80]. Collagen is often used to improve the cell proliferation and increase cell adhesion of biopolymers that are non-adhesive [74]. For example, in agarose-collagen hydrogels, the presence of collagen would soften the scaffold and allow for the growth of spheroids over longer times [68]. The diffusion of nutrients and drugs is also faster with higher ratios of collagen. This is explained by the morphology of the scaffold having more pores with larger sizes when increasing the amount of collagen.

### 3.2. Gelatin

Gelatin is obtained from the partial hydrolysis of collagen. Amide groups are hydrolyzed into carboxyl groups, and depending on the process, different carboxyl group densities can be obtained [28]. Type A gelatin is obtained with the acid hydrolysis of collagen and results in positively charged gelatin with a low density of carboxylic groups. Type B gelatin is formed when processed in basic conditions; it has a large number of carboxylic groups and a negative charge at a neutral pH [23].

Gelatin forms gels at low temperatures but has low viscosity at body temperature. With decreasing temperatures, a triple helix structure is formed that increases the viscosity and modulus of the gelatin. This structural change is reversible, and the thermoresponsive viscosity of gelatin is a significant advantage for 3D printing.

Due to the presence of amino acids residues and cell adhesion sites (RGD amino acid sequences), gelatin has been widely used to enhance cell attachment and proliferation [27]. However, it can degrade in physiological conditions and has weak biomechanical properties [65]. To overcome these limitations, it can be cross-linked or combined with polysaccharides. Gelatin can be modified with methacrylate groups to form gelatin methacrylate (GelMA), which is easily cross-linkable with UV light, providing high stability and printability. On the other hand, Jiang et al. added CNF as fillers in gelatin gels to improve their biomechanical properties [58]. The highest compression strength was obtained with 10% of CNF, and the final 3D printed scaffolds were cross-linked with genipin to increase the time stability [58].

Breast cancer cells have been grown in scaffolds containing gelatin. Increasing the amount of gelatin has been reported to increase the cell proliferation, leading to larger spheroids [27]. Scaffolds of GelMA were reported to sustain the growth of MDA-MB-231 and MDA-MB-468 breast cancer cells [82]. Hydrogels with a concentration of 10% of GelMa were found to be optimum as they had larger pores and a higher proliferation rate than 15% GelMA hydrogels and were strong enough to sustain cell growth compared to 5% GelMA hydrogel. Cells cultured in GelMA were also reported to be more invasive than similar cells cultured in 2D, characterized by the overexpression of the matrix metalloproteinase-2 (MMP2), vascular endothelial growth factor (VEGF), spectrin Alpha, Non-Erythrocytic 1 (SPTAN1), and transforming growth factor beta 1 (TGFβ1) genes, which are associated with invasiveness.

### 3.3. Silk Fibroin

Silks are proteins-based fibers that are spun by spiders and silkworms among others [83]. The amino acid sequence and hierarchical structure of silk depend on the species as well as the extraction and purification processes. Silk from *Bombyx mori* is produced at an industrial scale, and therefore, it is one of the most widely studied silks. Silk fibroin refers to the purified silk after removal of the sericin coating present in the cocoons. It has a semi-crystalline structure with β-sheet crystals that gives high mechanical strength to the fibers while embedded in an amorphous matrix. The β-sheets are cross-linked with inter and intra hydrogen bonds, which make the structure prone to rearrangement in the presence of water.

Silk fibroin solutions have a low viscosity; hence, it is challenging to form stable 3D printing constructs [84]. To improve the printability, high concentrations or additives are used. However, increasing the concentration of fibroin solutions usually requires complex processes that damage the protein structure. The addition of more viscous polymers, such as gelatin, improve the printability, and at the same time, limit the brittleness and low flexibility of silk fibroin [65]. The cross-linking of silk fibroin can be done mechanically and chemically, for example, with sonication to induce β-sheet crystallization or enzymatically with tyrosinase enzymes [65,84].

The advantages of using silk fibroin as cancer cell tissue models are its relatively high strength (0.1 to 1 MPa) and good cell adhesion property [85]. Talukdar et al. compared cancer cell adhesion between silk fibroin from *Antheraea mylitta* and *Bombyx mori* and found improved cell adhesion on a scaffold from *A. mylitta* compared to *B. mori* due to the presence of the RGD tripeptide motif in *A. mylitta* fibroin [86]. The *A. mylitta* fibroin had cell adhesion and viability comparable to Matrigel for the growth of MDA-MB-231 cells. Silk fibroin can also be used to improve and tune the mechanical properties of 3D scaffolds. For example, when silk fibroin was added to a chitosan scaffold, it allowed the increase of the compressive modulus of chitosan to 0.6 MPa and reduction of the degradation rate of chitosan due to the chemical cross-linking between the carboxyl groups of silk fibroin and the amino groups of chitosan [73].

### 3.4. Alginate

Alginate is a naturally occurring linear anionic polysaccharide produced by marine brown algae [87] and some bacterial species [88,89]. Alginate is composed of 1,4-linked β-d-mannuronic acid (M), and its C-5 epimer α-l-guluronic acid (G). The content and distribution of M-, G-, and polyalternating MG-blocks depend on both the alginate source and the growth conditions [90]. G-blocks interact strongly with some divalent cations (interactions are stronger with Ba^2+^ followed by Sr^2+^, Ca^2+^, and Mg^2+^, respectively) and form ionic cross-links [91]. Polyelectrolyte complexes with cationic polymers (for example, poly-lysin) can also be formed to form alginate gels [92]. The mechanical properties of alginate hydrogels depend on the alginate composition, distribution, molecular weight (MW), and the cross-linking ion.

Alginate is suitable for 3D printing applications where cross-linking with divalent cations can be used to form hydrogels. The viscosity of alginate-based inks can be tuned by varying the alginate concentration, the ionic strength of the solvent, and the MW [93,94]. Concentrations between 8% and 12.5% have been considered optimal for 3D printing scaffolds for breast cancer tissue models [24]. At concentrations below 8%, filaments were not formed while at concentrations above 12.5%, the adhesion between filaments was poor.

**Figure 5:**
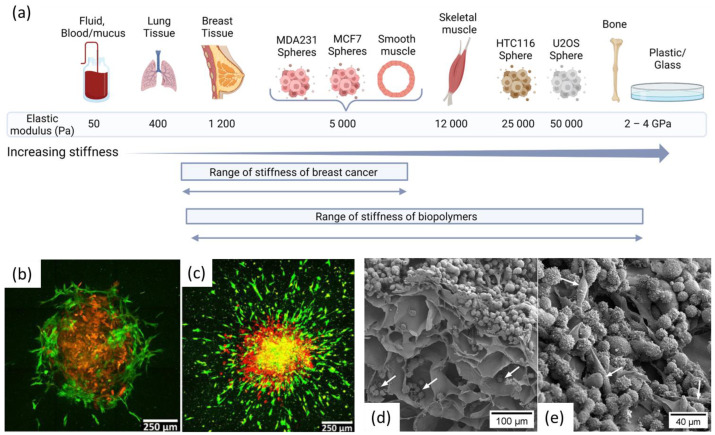
(**a**) Elastic modulus of various tissues, including normal breast tissue and breast cancer tissue. Data extracted from [22,96] and figure created with BioRender.com b. and c. Tumor spheroid invasion in 3D gel containing only collagen (**b**) and alginate and collagen (**c**) A spheroid transferred into a 3D gel at day 0 and cultured for 6 days in the gel shows minimal invasion only of human mammary fibroblasts cells in collagen and extensive invasion in alginate-collagen gel. Red color illustrates MDA-MB-231 cells expressing mKate fluorescent protein and green color illustrates human mammary fibroblasts cells expressing green fluorescent protein. Reproduced with permission from [95]. (**d**,**e**) MDA-231 cells growing on nanocellulose scaffolds. d. Cross-sectional image of a 3D scaffold. Note the cells reaching the pores of the scaffold (white arrows). (**e**) A heterogeneous population of MDA-231 cells (round and elongated cells indicated by white arrows), growing on the surface the scaffold.

Alginate has been used in several studies as a scaffold for breast cancer cell growth [24,27,43,69,75,95]. However, alginate does not have any cell adhesive sites, which is the reason why it is often used in combination with other bioactive polymers, such as gelatin or collagen. Jiang et al. 3D printed alginate/gelatin inks where alginate provided viscous properties during printing and mechanical support after cross-linking while gelatin provided elastic properties and bioactivity to promote cell adhesion [32]. Alginate-chitosan 3D scaffolds were found to promote cancer stem cell enrichment compared to 2D substrates [69]. Liu et al. demonstrated that the presence of alginate in alginate-collagen hydrogels increased the porosity of the network so that spheroids not only grow, but also migrate into the scaffold [95]. They managed to reproduce the follow-the-leader migration pattern observed in vivo where fibroblasts lead the invasion followed by cancer cells (Figure 5b,c).

### 3.5. Chitosan

Chitin is the second most abundant biopolymer found on Earth. It is found predominantly in the exoskeleton of insects and crustaceans and in the cell wall of fungi and yeast [97]. Chitin is a homopolymer composed of β-1,4-linked *N*-acetyl glucosamine (GlcNAc), and chitosan can be obtained by partial de-*N*-acetylation of chitin. Chitosan is generally described by its degree of acetylation, D_A_ [98]. The D_A_ largely dictates the properties of the chitosan, including solubility, pH sensitivity, and shape in solution. The amino group at C2 with a pKa of approximately 6.5 is responsible for the polyelectrolyte properties of chitosan [99]. The polyelectrolyte properties of chitosan are exploited when chitosan is used in a 3D printing system. When the pH is increased above the pKa, chitosan transitions from a soluble semi-rigid random coil in the solution to an insoluble polymer upon neutralization [100]. Both the degree of acetylation and the MW influence the solution properties of chitosan. The good printability of chitosan whilst still obtaining hydrogels with satisfying mechanical properties has been reported by several authors for MWs from 180 to 250 kDa and an D_A_ from 0.1 to 0.2 [101,102]. For the ink, the solvent used is commonly an acid or a mixture of acids, and the precipitation is done by increasing the pH. Wu et al. [102] prepared chitosan inks with an 8% chitosan solution (*w*/*v*) with MW 207 kDa and D_A_ 0.1. The solvent used was a mixture of 40% acetic acid, 10% lactic acid, and 3% citric acid. The resulting hydrogel was used to obtain guided cell growth, largely attributed to the mechanical and surface properties of the hydrogel.

Different strategies have been explored to stabilize 3D printed chitosan structures. Ionotropic chitosan-based hydrogels can be obtained with cross-linking using a small, negatively charged molecule; the most commonly used is tripolyphosphate/sodium tripolyphosphate [103]. Charge-to-charge interactions are also the basis for polyelectrolyte complexes formed between two polymers bearing opposite charges (Figure 3). Several polyanions have been explored for the preparation of bioinks with chitosan, including alginate, dextran sulfate, pectin, and chondroitin sulfate [104,105,106,107,108]. When using this approach, the mechanical properties of the hydrogel depend on the nature of both biopolymers as well as the method used for mixing. Irreversible cross-linking using small spacers can also be used to obtain chitosan hydrogels. The method leads to hydrogels with improved mechanical properties as well as a decreased biodegradation rate [109].

In a recent study by Taira et al. [78], electrodeposition-based printing was used to print a chitosan (D_A_ 0.2)/gelatin hydrogel. Human breast cancer cells (MCF-7) were successfully encapsulated and proliferated inside the hydrogel. In a similar study of MFC-7 proliferation, Mohammadi et al. [110] demonstrated the 3D printing of a hydrogel based on chitosan and carbon dots for the detection of microRNA-21, which is a biomarker for early-stage breast cancer. The adhesion and proliferation of MFC-7 has been shown to depend on the surface properties of the scaffold, where increased swelling resulted in improved adhesion. It follows that low D_A_ promotes MFC-7 adhesion.

### 3.6. Hyaluronic Acid

Hyaluronic acid (HA) is a glycosaminoglycan sometimes referred to as hyaluronan, as it is present as polyanion in the ECM. It is a linear anionic polysaccharide that is water soluble at the physiological pH. It is found in different connective tissues where it has structural and functional roles [28]. It has been used in biomedical applications, such as cell encapsulation, skin regeneration, tissue protection, and drug carrier, among others [28].

Due to its high-water absorption capacity, HA forms hydrogels. However, these hydrogels are highly viscous and have long gelation times. Because its gelling properties are not suitable for 3D printing, it has poor mechanical properties [72], and it has a fast degradation rate, HA cannot be used alone as a scaffold. Instead, HA is often combined with polysaccharides, such as chitosan, alginate, and CNF [72,111,112], or is chemically modified [57,64]. For example, the modification of HA with 4-pentenoic anhydride allowed the photoinduced cross-linking of the printed hydrogel to maintain its shape stability over time [64]. Moreover, changing the MW from 1 MDa to 1.5 MDa increased the viscosity of HA thus printing with high MW HA could be done at a lower concentration [64].

HA is present in high concentrations in the ECM surrounding tumors and favors tumor progression [113]. The presence of HA in cancer tissue models can mimic the cell-hyaluronic acid interactions that promote cell proliferation, differentiation, and migration. HA interacts mainly with cell-surface receptors CD 44 and RHAMM (receptor for hyaluronic-acid-mediated motility) [114]. Suo et al. synthesized hydrazone cross-linked HA hydrogels from two HA derivatives [115]. After showing adapted mechanical properties and high viability of MCF-7 cells, they evaluated the expression of three growth factors. The expression levels of vascular endothelial growth factor (VEGF), basic fibroblast growth factor (bFGF), and interleukin 8 (IL-8) were upregulated in 3D culture of MCF-7 cells and higher than in 2D models. bFGF and IL-8 improve cell survival while VEGF promotes metastasis and angiogenesis. HA has also reduced cell adhesion, which promotes cell–cell interactions and favors the creation of cellular aggregates and tumor spheroids [113].

### 3.7. Cellulose Nanofibers

Cellulose, the most abundant natural polymer on earth, is probably one the of last biopolymers to enter the additive manufacturing space for biomedical applications. The conventional source of cellulose fibers is wood, but alternative sources like bagasse, flax fibers, cotton, algae, and tunicate are emerging [116,117].

#### 3.7.1. CNF from Wood

Cellulose fibres are composed of a linear polysaccharide composed of β(1→4) linked d-glucose units. Hydrogels can be obtained from cellulose fibers when the fibers are deconstructed to the nanosize (i.e., CNF) [118]. Oxidation with NaClO and mediated by 2,2,6,6-Tetramethylpiperidinyloxy (TEMPO) is one of the most common pre-treatments. The TEMPO pre-treatment oxidizes the primary alcohol of the anhydroglucose unit into carboxylic acid, creating negative charges and, thus, repulsive forces between the fibrils. This facilitates the deconstruction of the cellulose fiber wall into CNF; nanosized cellulose fibrils with high aspect ratio and diameters under 20 nm and lengths around 1000 nm are then obtained [119]. TEMPO CNF from wood has proven to be ultrapure and appropriate as a biomaterial for biomedical applications [120]. Other pre-treatments, such as phosphorylation, periodate oxidation, and carboxymethylation, are used to introduce surface charges and modify the nanofibers [121]. Carboxylated CNF has several advantages for 3D printing, such as enabling ionic cross-linking or avoiding the clogging of the nozzle due to ionic repulsive forces. Concentrations between 1 and 5 wt% were reported optimal for the 3D printing of CNF [41]. At a low shear, CNF gels present a storage modulus much higher than their loss modulus, typical of strong gels. Moreover, their viscosity decreases linearly with an increasing shear rate (shear-thinning behavior), which is a major advantage for 3D printing by micro-extrusion.

Only a few studies have reported the preparation of a CNF scaffold for cancer cell growth [25,42,62,122] (Figure 5d,e). In cancer tissue modelling, CNF are used as a structural component of the scaffold as their porosity and fibrous topography are similar to the extracellular matrix [41]. A reported compressive modulus for 3D printed CNF based scaffold are in the range of 1–10 kPa [59,62,123], which is suitable for the growth of breast cancer cells [96] (Figure 5a). Other parameters, such as a surface charge of the CNF, could influence cells adhesion and proliferation. Liu et al. studied the growth of HeLa cells in TEMPO oxidized CNF scaffolds of different surface charge densities [122]. Lower charge density was reported to improve cell viability and growth. Structural changes were also reported, and it was difficult to tell if the differences in cell proliferation were only due to the charge content or also to the physical properties.

#### 3.7.2. CNF from Macro-Algae

Cellulose and alginate are present in the cell wall of macroalgae, from which they can both be extracted [124]. This is most interesting, as both cellulose and alginate are two polysaccharides for 3D bioprinting. Provided an appropriate biorefinery approach, it is possible to extract CNF from alginate-extracted algae. However, the yield and purity can vary depending on the species and the extraction method [125]. Cellulose from algae is not bound to lignin like in plants; hence, the extraction methods can be milder than for cellulose from wood. However, other impurities, such as xylose (another monosaccharide that is commonly isolated from woody biomass), can be attached to the cellulose obtained from algae. Wahlström et al. prepared CNF from *Ulva lactuca*; the CNF contained 10–15% xylose [126]. They presented the same crystalline structure and thermal properties as CNF from wood.

#### 3.7.3. CNF from Tunicate

The tunicate is a marine animal that produces cellulose in the tissue that covers its entire epidermis [127]. The tunicate is an emerging source of cellulose that can be grown underwater and does not compete with land farming. CNF from tunicate have shown promising results for tissue engineering [128]. High purity Iβ cellulose can be extracted from tunicate. Cellulose extracted from tunicate has high crystallinity, leading to long individualized nanocrystals over 1 µm in length after hydrolysis [129].

#### 3.7.4. CNF from Bacteria

High purity CNF produced by bacteria forms a highly entangled network of nanofibers [130]. The water resistance of bacterial cellulose is interesting for biomedical application. However, due to the entangled structure of the nanofibers, they do not form 3D printable gels unless the bacterial CNF membrane is post-treated by, e.g., disintegration and homogenization [131]. Wang et al. studied MDA-MD-231 cell growth on freeze-dried scaffolds of bacterial CNF and bacterial CNF coated with cross-linked gelatin to introduce bioactivity [71]. Cells proliferated in larger numbers in the presence of gelatin, forming multilayered growth and cells clusters, which was not the case with pure bacterial CNF scaffold. On the other hand, the bacterial cellulose provided a great support network with improved mechanical properties and a high porosity for cell proliferation compared to gelatin alone.

## 4. Scaffolds for Mimicking Breast Cancer Tissue Microenvironment

Compared to 2D substrates where cells grow in monolayer, 3D scaffolds allow the cells to grow in multiple layers and spheroids, which more resemble the behavior of tumors in vivo [69]. To obtain such spheroids, specific structures with interconnected porous network are needed with two level of pore sizes. Submicron pores forming a percolating pattern are necessary for the diffusion of nutrients and oxygen while macropores of hundreds of microns allow the cells to attach, migrate, and proliferate [132]. In 3D scaffolds, oxygen and nutrients gradients can occur, creating different growth conditions within the scaffold. Murphy et al. studied the influence of 3D scaffold porosity on osteoblast cells adhesion and proliferation [133]. Pore size around 100 µm and high surface area were related to high initial cell adhesion but hindered proliferation of the cells inside the scaffold and led to accumulation of cells on the edges. In that case, pores of 300 µm and larger were considered optimal for long term proliferation of the cells [133].

Several factors can influence the porosity, such as swelling capacity, cross-linker amount, and hydrogel concentration [134]. For example, Liu et al. studied the porosity of alginate/collagen hydrogels depending on the CaCl_2_ concentration [95]. A CaCl_2_ concentration of 7.5 mM, which corresponded to a porosity of 93% and a median pore size of 95 µm, was considered optimal for MDA-MB-231 breast cancer cells proliferation. It should be kept in mind that porosity is closely linked to the mechanical properties of the scaffold; hence, tuning one of them influences the other, and it is difficult to study both factors independently [68].

### 4.1. Biomechanical Properties

Biomechanical properties of the 3D matrix can influence cell proliferation and differentiation. Parameters such as stiffness and elasticity should be adapted to the targeted tissue (see Figure 5a) [135]. The optimal stiffness of the matrix depends on the type of organ and if it is normal tissue or tumor tissue. In fact, the stiffness of tumors is usually higher than that of healthy tissues [136]. Different cancer cells also have different stiffness requirements. Redmond et al. listed the suitable stiffnesses for different kind of breast cancer cells [14]. Jabbari et al. studied the optimal elastic modulus for different cancer cells and found that a Young’s modulus between 2 and 25 kPa was preferred for MCF-7 and MDA-MB-231 cell lines. In comparison, a modulus of 50 kPa was optimum for U2OS osteosarcoma cells (Figure 5a) [96]. The modulus has a noticeable influence on the number of tumor spheroids, but also their size [27,68]. In comparison, conventional polystyrene 2D culture plates have a Young’s modulus around 2–4 GPa, and hence, their mechanical properties are not suitable for cancer cells cultures. There are different ways to measure the mechanical properties of a scaffold; micro, nano-indentation, and atomic force microscopy (AFM) are methods that assess the local characteristics that are relevant for, e.g., cell migration in a 3D printed scaffold [137]. Compression tests at the macroscale are more global and can give elastic modulus values that deviate by one order of magnitude. For precise characterization of the scaffolds, a local analysis is recommended in the same conditions as the cell study.

The use of 3D printing to design the scaffold allows us to tune the biomechanical properties and adapt to the cells by changing the concentration of the ink or the amount of cross-linker, for example. However, in 3D printing, tuning the biomechanical properties is closely related to the structure and architecture of the matrix, and it is hence difficult to only study the effect of matrix stiffness on cell proliferation [138]. Cavo et al. tuned the mechanical properties of an alginate gel by changing its concentration as well as the cross-linker concentration. Gels with stiffnesses between 150 and 4000 kPa, measured by an AFM, were obtained. Cell viability of MCF-7 was found to be inversely proportional to the gel stiffness, gels with elastic moduli between 150 and 200 kPa showed cell proliferation to cell clusters of 100 µm to 300 µm after one and two weeks, respectively [43]. Jiang et al. also demonstrated that the tumor spheroid formation varied with the mechanical properties of the hydrogels. Softer hydrogels with a Young’s modulus between 5.5 and 7.9 kPa showed larger and higher numbers of tumor spheroid formation than harder hydrogels with a modulus between 20 and 23 kPa [27]. Desired mechanical properties should be compatible with 3D printing parameters like ink viscosity and shape fidelity among others. Moreover, biomechanical properties of the scaffold can be influenced by the cell growth media and the time of cell proliferation as, for example, monovalent cations present in the growth media could replace divalent cations in ionically cross-linked scaffolds, which would weaken the structure over time [25].

### 4.2. Bioactive Surface for Cell Adhesion, Proliferation, and Differentiation

In vivo, the extracellular matrix (ECM) forms the scaffold of organs and tissues and, thereby, the 3D microenvironment and growth platform for the cells. The structure of the ECM differs between organs, but it is mainly composed of different collagens, heparin, laminins, glycosaminoglycans (GAGs), elastin, and fibronectin [22]. The ECM composition and general microenvironment can be heterogenous within a tumor. One single tumor can have sections that are very soft and porous, whereas another part of the tumor can be hard and bone-like. This affects the cells since the microenvironment constituents regulate tumor growth, invasion, and metastasis [139,140]; hence, it is important to develop scaffolds that functionally mimic the ECM. The 3D printing of scaffolds has the possibility to mimic these properties with the possibility of printing different materials simultaneously, and it also has the possibility to design gradients within the printed scaffolds. Both biomechanical properties and the chemistry of the scaffold influence the cell characteristics, such as adhesion, differentiation, proliferation, epithelial-mesenchymal transition (EMT), and cancer stemness. A lack of adhesion promotes cell-to-cell interactions and spheroid formation [113] and lack of surrounding ECM in a monolayer cell culture disrupts fundamental cellular processes, such as a matrix invasion, linked to metastasis [53,141].

To improve cell adhesion, polysaccharides have been mixed with collagen, gelatin, or fibroin [142]. The addition of the RGD binding sequence to collagen-like proteins significantly increased the adhesion of the cells, enabling their proliferation [142]. Additives like proteins present in the tumor microenvironment could also be added to the ink formulation to better mimic the tumor microenvironment [24]. However, collagen, gelatin, and alginates are difficult to 3D print due to their low viscosity, and their structures are not stable in biological conditions over long periods of time [28]. On the other hand, only a few studies have reported on CNF for the fabrication of scaffolds to model the cancer microenvironment [25,143]. A CNF is durable (not biodegradable by human cells), provides structural stability to the construct, and has a 3D pore network that facilitates cell proliferation [41]. Preliminary works successfully demonstrated the suitability of a CNF scaffold for cancer cell growth; however, it was not representative of the complexity of the tumor microenvironment. The addition of bioactive entities that will favor the cancer growth is needed. The bioactivity of structural CNF can be introduced with the grafting of RGD-motifs (Arg-Gly-Asp-containing peptides that promote cell-adhesion) [144].

### 4.3. Biological Components to Optimize Breast Cancer Tissue Models

In addition to the structural and surface characteristics mentioned above, there are two aspects that are important to consider when biofabricating functional tissue models to replicate the tumor microenvironment: (i) extracellular vesicles (EVs, lipid bilayer particles for transportation of bioactive molecules) and (ii) signaling proteins. The role of EVs to modulate the signaling between cells and the tumor microenvironment in the development of cancer has recently gained attention [145]. It has also been demonstrated that EVs contribute to the increase of drug resistance of cancer cells [146]. On the other hand, signaling proteins have been mentioned regarding the vascularization of scaffolds and tissue models [147].

Biofabrication by 3D printing could be a potent method to mimic the gradients of EVs, chemicals, and signaling proteins naturally encountered during tumor tissue development. Such an approach is relevant, although demanding to implement, and would require identifying the concentrations of the signaling proteins that are most attractive for different cancer cells within the same scaffold. These will then differ between cancer types and also between sub-groups within the same cancer type. We envisage three approaches that could be applied in this respect; (i) the biomaterial-based encapsulation of signaling proteins (mimicking EVs) to be included in bioinks, (ii) the binding of the proteins directly to the biopolymers in the ink, and (iii) the addition of the components directly to the medium used for the 3D models.

Cancer cells mature and change to a new phenotype over time; sometimes up to 3 weeks are needed to allow cells to adjust to their new microenvironment [148]. Nanocellulose has an advantage in this case, as nanocellulose is not biodegradable in these conditions and forms structurally stable scaffolds. Stable and mature cancer tissue models are relevant for testing drugs for approximately 48 h [24]. However, optimal 3D printed breast cancer tissue models for drug testing should be stable over additional time, especially if the models include infiltrating cells since these cells will be less accessible compared with cells grown at the surface.

## 5. Molecular Profiling of 3D Cultured Cells

**Figure 6:**
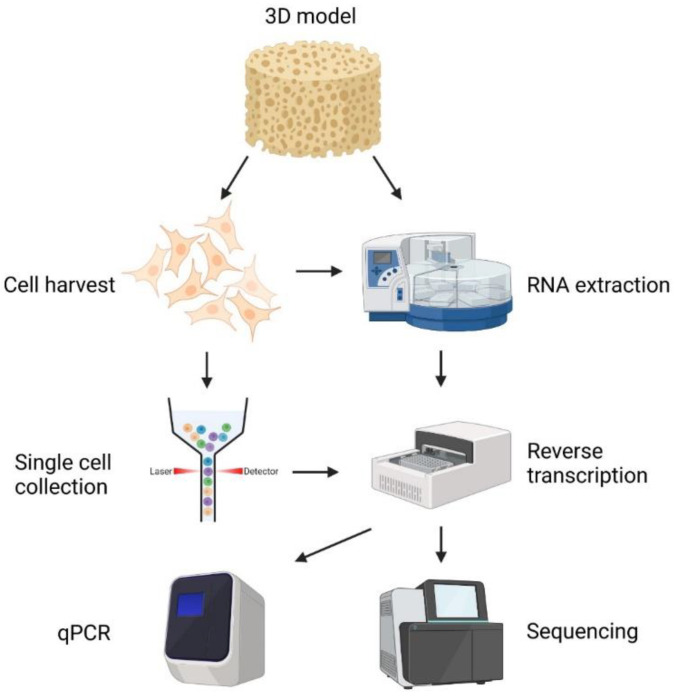
Schematic overview of gene expression profiling using 3D models. To analyze the gene expression profile of all cells, RNA is extracted from either the entire 3D model or the harvested cells followed by reverse transcription and either qPCR or sequencing. Direct lyzed cells from the scaffold can also be transferred directly to the reverse transcription step without any extraction [155]. For single cell gene expression profiling, individual cells need to be collected from the 3D model followed by reverse transcription and either qPCR or sequencing. Created with BioRender.com.

There are several approaches to study 3D cultured cells, including both cellular and molecular profiling. Gene expression profiling is one of the most powerful tools to assess molecular properties of cells cultured in 3D models. The whole transcriptome can be analyzed using RNA sequencing, while the expression of specific marker genes often is profiled by reverse transcription quantitative real-time polymerase chain reaction (RT-qPCR). Typically, all cells in the experimental system are assessed, but it is also possible to analyze individual cells to delineate the molecular properties of different cell types and their cellular states. To obtain reliable data, an optimized experimental workflow is needed, including the following steps: cell collection, RNA extraction, mRNA analysis, and data analysis (Figure 6).

Today, there are established guidelines for RT-qPCR that should be followed for accurate gene expression profiling [149] while the overall recommendations for RNA sequencing are less standardized. To assess the properties of breast cancer cells, a comprehensive strategy is to quantify the expression of well-characterized marker genes related to known cellular functions, such as cell proliferation, differentiation, drug resistance, epithelial-to-mesenchymal transition, and stemness. To generate reliable data, it is usually advantageous to analyze multiple markers in each group. This approach has been successfully applied to different 3D models cultured with breast cancer cell lines [24,25], demonstrating that several processes are significantly regulated compared with 2D monolayer culture systems. A promising approach for 3D cell culture models is to use them as a drug screening platform since the cell’s characteristics are better at mimicking the human in vivo situation compared with 2D cell cultures. A patient-derived scaffold from breast tumors has been shown suitable as a tool to monitor chemotherapy responses in human tumor microenvironments [54]. It has also been demonstrated that breast cancer cells grown in 3D printed scaffolds treated with the chemotherapy drugs doxorubicin or 5-Fluorouracil responded more similarly with cells grown in tumor tissue scaffolds compared with monolayer cultured cells, as analyzed by changes in gene expression of marker genes before and after treatment with different concentration of the drugs [24].

A clear benefit of using 3D models is that cancer cells can find or create their own microenvironment. This feature enables multiple cellular phenotypes with a different degree of cellular activation. A standard analysis of all cells together do not provide information about all cell types and cell heterogeneity. To address these limitations, individual cells can instead be profiled. First, intact cells need to be harvested from the scaffold followed by single cell collection and gene expression profiling. Individual cells can be collected by different methods, such as fluorescence-activated cell sorting (FACS), *microaspiration*, and droplet-based microfluidics. Today, there is a plethora of commercially available techniques that are suitable for a single-cell RNA analysis. Both RT-qPCR [150] and RNA sequencing [151] are standardized approaches to profile the expression of genes in single cells, and numerous approaches exist for sequencing [151]. Transcription occurs in bursts, resulting in highly variable expression levels in seemingly homogenous cell populations [152,153]. The mean expression of most genes is also very low, where most cells do not display any transcript for lowly expressed genes. Consequently, numerous cells, sometimes >> 10,000 cells, need to be analyzed to characterize the entire cell population in detail, especially when multiple cell types are expected. Compared with cell population analysis, a single cell analysis requires highly optimized workflows that minimize molecule losses. Importantly, spatial transcriptomics have recently been developed that provide gene expression profiling with single-cell resolution, keeping the spatial information about each cell’s position in the microenvironment [154].

## 6. Conclusions and Perspectives

This review has focused specifically on breast cancer tissue models. However, the assessment can be considered valid for most solid tumor types despite their difference in cellular characteristics and microenvironments. It seems to be the consensus that 3D structures provide a better representation of the cells’ microenvironment than 2D cell growth and allow multilayer cell growth. Drug efficiency is also decreased in 3D, which provide results closer to in vivo tests. Different compositions of hydrogels have been proven efficient for cancer cell growth in 3D, such as gelatin/alginate, collagen/alginate, chitosan/gelatin, and silk fibroin/chitosan. Nanocellulose was also emphasized as a relatively recent biomaterial for 3D printing and for biofabrication.

Three-dimensional printing has proven to be an excellent technology that allow reproducibility of the process to form scaffolds for cell seeding and could be further utilized to study scaffold structure (porosity, mechanical properties, composition) influence on cell growth and tissue formation. A comparison between drug efficiency on a 3D printed scaffold and in vivo is also key to further develop cancer tissue 3D models. Three-dimensional printing will also facilitate the deposition of components at pre-determined locations in a 3D model to biofabricate microenvironments similar with growth conditions in human tumors. This is most critical and will require the optimized combinations of various components and processes, such as (i) 3D printable biopolymer blends with RGD cell attachment sites and tailored for the target tissue, (ii) feasible and cell-friendly cross-linking approaches, (iii) signaling molecules that may be encapsulated for controlled delivery during cell culturing, and (iv) optimized biofabrication technology to secure adequate porosity to allow for gradients of oxygen and nutrients that are necessary for cancer tissue development. This is expected to secure 3D cell culture systems, mimicking the microenvironment surrounding the cancer cells. Further, we expect that spatial transcriptomics in combination with 3D models will be most useful to study cellular phenotypes, especially when evaluating the effects of different scaffold components used in 3D models and the corresponding relation to cell behavior.

## Data Availability

Not applicable.
